# Exploring the multifactorial aspects of Gut Microbiome in Parkinson’s Disease

**DOI:** 10.1007/s12223-022-00977-2

**Published:** 2022-05-18

**Authors:** Sujith Pavan, Arvind N. Prabhu, Sankar Prasad Gorthi, Bhabatosh Das, Ankur Mutreja, Vignesh Shetty, Thandavarayan Ramamurthy, Mamatha Ballal

**Affiliations:** 1 Enteric Diseases Division, Department of Microbiology, Kasturba Medical College, Manipal Academy of Higher Education, Manipal, Karnataka India; 2 Department of Neurology, Kasturba Medical College, Manipal Academy of Higher Education, Manipal, India; 3 Department of Neurology, Bharati Vidyapeeth Medical College & Hospital, Pune, India; 4 Molecular Genetics Laboratory, Centre for Human Microbial Ecology, Translational Health Sciences and Technology Institute, NCR Biotech Science Cluster, Faridabad, India; 5 Department of Medicine, University of Cambridge, Cambridge, UK; 6 National Institute of Cholera and Enteric Diseases, Kolkata, India

**Keywords:** Gut-Brain Axis, Gut dysbiosis, Microbial Metabolites, Microbiome, Parkinson’s Disease

## Abstract

Advanced research in health science has broadened our view in approaching and understanding the pathophysiology of diseases and has also revolutionised diagnosis and treatment. Ever since the establishment of Braak’s hypothesis in the propagation of alpha-synuclein from the distant olfactory and enteric nervous system towards the brain in Parkinson’s Disease (PD), studies have explored and revealed the involvement of altered gut microbiota in PD. This review recapitulates the gut microbiome associated with PD severity, duration, motor and non-motor symptoms, and antiparkinsonian treatment from recent literature. Gut microbial signatures in PD are potential predictors of the disease and are speculated to be used in early diagnosis and treatment. In brief, the review also emphasises on implications of the prebiotic, probiotic, faecal microbiota transplantation, and dietary interventions as alternative treatments in modulating the disease symptoms in PD.

## Introduction

Parkinson’s disease (PD) is the second most common neurodegenerative disorder, Alzheimer’s disease being the most common. PD afflicts approximately ten million people globally (Statistics | Parkinson’s Foundation 2021; Kaur et al. 2019). PD’s prevalence and incidence increase with the advancing age, and the incident rate is higher in men (Statistics | Parkinson’s Foundation 2021). It has been two centuries in the wake of a prologue to PD with an observational essay on the ‘Shaking Palsy’ by James Parkinson (Parkinson 2002; Jost and Reichmann 2017), the investigation to identify the trigger that prompts the disease is at its prime. However, since Lewy discovered the eosinophilic inclusion body and established the involvement of alpha-synucleinopathy, researchers have uncovered most aspects of PD pathophysiology and have described it as a multifactorial disease. Environmental factors, exposure to chemicals (insecticides, pesticides), xenobiotic toxins, genetic predisposition, altered dopamine metabolism, mitochondrial dysfunction, oxidative stress, neuroinflammation, and aging play a crucial interactive role in the pathogenesis of PD (Riess and Krüger 1999; Kaur et al. 2019; Pang et al. 2019).

PD’s key cardinal motor features include resting tremors, rigidity, slowness of movement, and gait disturbance (Kasper et al. 2014). Most PD patients start experiencing non-motor symptoms (NMS) markedly decades before the motor symptom surfaces (Poewe 2008; Heintz-Buschart et al. 2018). Gastrointestinal (GI) symptoms such as dry mouth, constipation, and defecatory dysfunction are highly reported NMS prior to the onset of motor symptoms (Cersosimo et al. 2013). Most of these NMS are overlooked during this prodromal stage. Treatment for PD is initiated only when the motor symptoms appear; by then, more than fifty percent of the dopaminergic neuron might have degenerated in the substantia nigra (Cheng et al. 2010).

Recent advancements in research demonstrated gut dysbiosis could initiate the neurodegenerative process in PD and serve as potential biomarkers for diagnostics and treatment modulation (Keshavarzian et al. 2015; Scheperjans et al. 2015). This review is structured to address the involvement of gut microbiota and its metabolites associated with intestinal integrity, genetic predisposition, motor and non-motor aspects, antiparkinsonian treatment, and diet in PD by considering the available global data.

## Alpha-synucleinopathy

Alpha-synuclein **(**α-syn) is a monomeric protein of 140 amino acids encoded by the SNCA (synuclein alpha) gene (Stefanis 2012). α-syn is found in small quantities in the heart, muscles, and other different tissues; however, it is abundantly found in the brain, especially in the presynaptic terminal tips of the neurons (Moons et al. 2020). In neurons, α-syn plays a role by inhibiting the release of neurotransmitters when over-expressed (Bendor et al. 2013). α-synucleinopathy is the confirmational change of the soluble protein α-syn into a pathological oligomeric beta-sheet structure (Meade et al. 2019) that loses its membrane binding capacity (Pang et al. 2019) and aggregates in the cytoplasm of neurons, glial cells, or nerve fibers, disrupting the cellular homeostasis inducing cytotoxicity (Luna and Luk 2015).

A few studies have demonstrated that α-syn can induce microglia or monocytes to produce proinflammatory cytokines (TNF-α and IL-1β) (Lee et al. 2010; Couch et al. 2011). Likewise, a study by Stolzenberg et al. reports a positive correlation between intestinal inflammation and expression of α-syn and observed α-syn to exhibit immune-modulatory or chemo-attractive properties that induce migration of neutrophils and monocytes and stimulate dendritic cells maturation (Stolzenberg et al. 2017). Hence these findings suggest α-syn is a component of the innate immune response of the GI enteric nervous system (ENS), and excessive exposure to α-syn can initiate PD (Stolzenberg et al. 2017). Immunohistochemical analysis of tissues of the spinal cord and peripheral nervous system by Beach et al. have divulged the high occurrence of phosphorylated α-syn in paraspinal sympathetic ganglia, the vagus nerve, and the GI tract of PD subjects (Beach et al. 2010). Similarly, α-syn has been demonstrated in the neurons of sigmoid mucosal samples collected two to five years before the onset of PD (Keshavarzian et al. 2015).

Holmqvist et al. verified Braak’s hypothesis by demonstrating the retrograde transfer of α-syn from the ENS to the brain in an animal experimental model. They injected different forms of α-syn obtained from human PD brain lysate and a recombinant α-syn into the intestinal walls of mice to demonstrate their transport via vagal nerve to the brain (Holmqvist et al. 2014). α-syn can directly activate microglia to upregulate Toll-like receptors (TLRs) and proinflammatory cytokines by serving as endogenous damage-associated molecular patterns (DAMPS). TLR signalling induces nuclear factor kappa B (NF-κB) activation, which is vital to dopaminergic neuron apoptosis. Microgliosis also increases nitric oxide production and further promotes α-syn pathology by inducing nitration of α-syn in neighbouring neurons and cell death (Béraud et al. 2011).

**Fig. 1:**
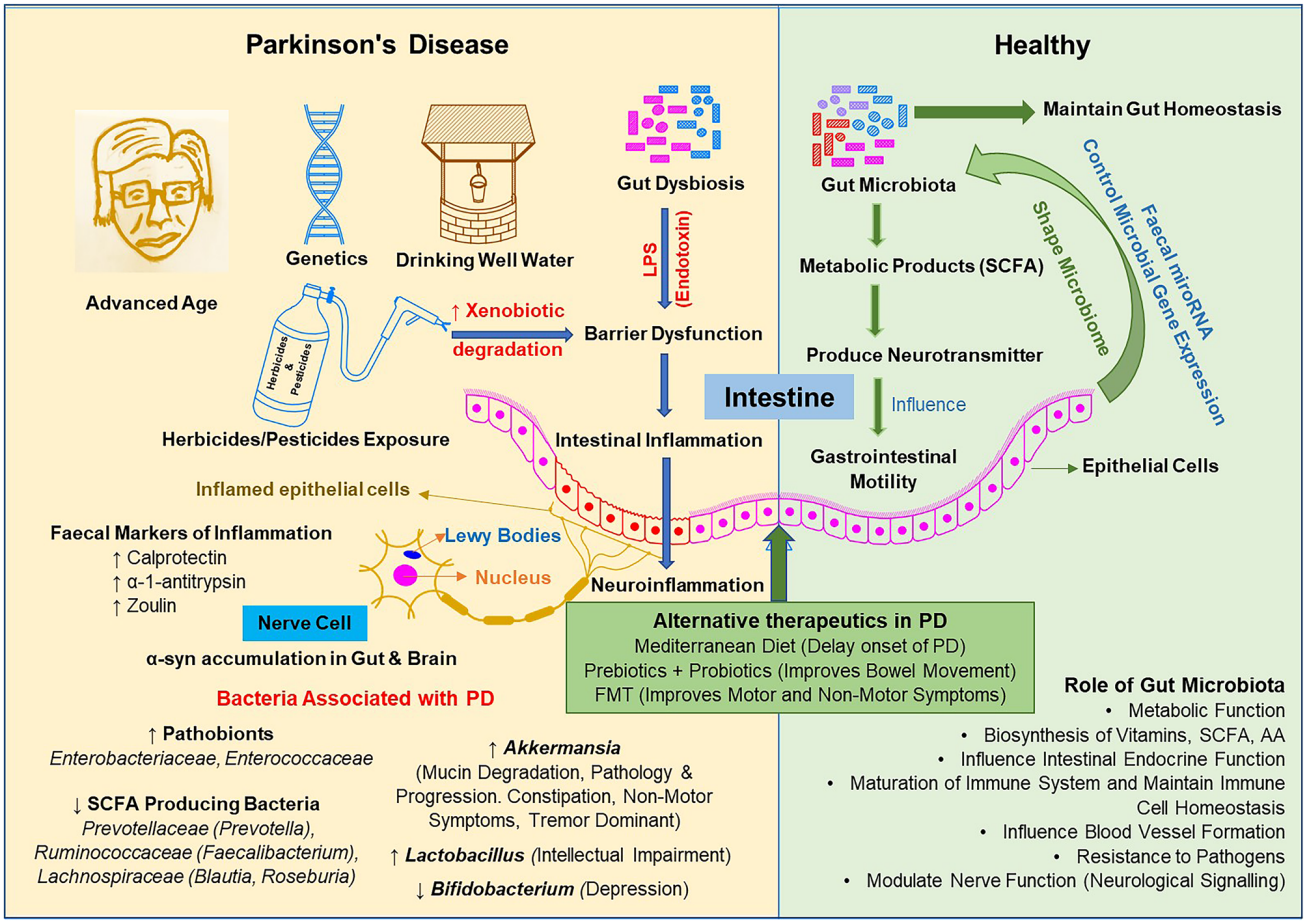
Schematic diagram summarizing the factors associated with PD. Abbreviations: LPS- Lipopolysaccharides, FMT- Faecal microbiome transplantation, SCFA- Short-chain fatty acids, AA- Amino acids, α-syn- alpha synuclein

Drinking well water and exposure to herbicides and pesticides in a farm or cultivation setting have been associated with increased risk factors in PD (Fig. 1) (Gatto et al. 2009; Freire and Koifman 2012). Hill-Burns et al. reported an increased xenobiotics degradation pathway, specifically for herbicides and pesticides in the PD patient’s gut (Hill-Burns et al. 2017). An animal model also demonstrates that exposure to such compounds can cause cell death of dopaminergic neurons and lead to movement disorder in mice (Blesa et al. 2012). Forsyth et al. hypothesised that the dysbiosis and exposure to bacterial endotoxin possibly initiate α-syn misfolding by prompting GI inflammation and hyperpermeability in PD patients (Forsyth et al. 2011). Researchers have also observed that exposure to bacteria-producing amyloid protein curli can induce α-syn accumulation in the gut and brain of mice (Chen et al. 2016). In addition, exposing mice to environmental toxins such as pesticide rotenone triggered the release of α-syn of enteric neurons into the extracellular matrix. These α-syn are shown to be taken up by presynaptic neurons or other non-neuronal cells, proving the transneuronal retrograde movement of α-syn in tissue culture study (Pan-Montojo et al. 2012).

## Microbiome, blood brain barrier and gut-brain communication

The microbiome comprises of bacteria, viruses, fungi, archaea, protozoa, and bacteriophages in their natural habitat. The human body is inhabited by 10–100 trillion bacterial cells, which outnumber the human cells by 1–10 folds (NIH Human Microbiome Project defines normal bacterial makeup of the body | National Institutes of Health (NIH) 2012); however, the number of bacterial and human cells are debatable. According to Sender et al., the human body contains roughly the same number of bacterial cells as human cells (i.e., the body contains 3.8 × 10^13^ bacteria and 3 × 10^13^ human cells) (Sender et al. 2016). Microbial community varies in diversity and composition at different body sites. The human gut harbours a vast majority of the microbial species encoding unique genes more than the human genome (Ley et al. 2006). Gut microbial diversity mostly remains stable in an individual through adulthood, maintaining a core microbiome unless there is a significant change in dietary habits. A dynamic shift in microbial diversity and composition is noted with debilitating immune system among the elderly as a health consequence (Dinan and Cryan 2017).

The Blood–Brain Barrier (BBB) develops during the foetal stage, and it is composed of multiple cell components: capillary endothelial cells, tightly sealed junctions, basement membrane (fibrous matrix), neuroglial membrane, glial podocytes (projection of astrocytes), and pericytes (Braniste et al. 2014), which work in concert to protect the central nervous system (CNS) by limiting the entry of various substances. Braniste et al. have defined the primary role of the gut microbiota in influencing the BBB during the early stage of development in their work on germ-free mice. This study has reported that BBB was highly permeable in germ-free mice, which continued to adulthood and had reduced tight junction protein expression compared to mice with normal intestinal microbiota (Braniste et al. 2014). Short-chain fatty acids (SCFA) neuroactive metabolites, like butyrate, a fermentation product of gut microbiota, can regulate tight junction protein expression and influence histone deacetylase activity of BBB epigenetically (Al-Asmakh and Hedin 2015; Hasan Mohajeri et al. 2018).

The bidirectional communication between the gut and the brain exists via the central vagal nerve and systemic metabolic routes (Hasan Mohajeri et al. 2018). Likewise, microbiota-gut-brain bidirectional interaction occurs through neural, endocrine, immune, and humoral networks (Carabotti et al. 2015). ENS innervates the GI system and is in close proximity to the intestinal lumen, which offers a vast area for significant interaction with microorganisms (Cosma-Grigorov et al. 2020). Faecal micro-RNA of the intestinal epithelial cells has been proposed to enter the gut microbial cells and control gene expression, eventually shaping the microbiome (Liu et al. 2016).

The influence of gut microbial colonisation on intestinal sensory-motor function has been demonstrated in experimental animal models exhibiting delayed gastric emptying and intestinal transit in germ-free mice (Carabotti et al. 2015). Furthermore, gut microbiota may impact the production of neurotransmitters like gamma-aminobutyric acid, and their metabolic by-products like butyrate promote the functioning of the nervous system (Bienenstock et al. 2015). In addition, bacterial fermentation product SCFA may control microglial by influencing its maturation and function in the CNS and maintaining homeostasis (Erny et al. 2015). Besides, bacterial SCFA-butyrate regulates the biosynthesis of serotonin (Yano et al. 2015), which is responsible for initiating peristalsis (Sikander et al. 2009), and thus gut microbiota influence GI motility (Yano et al. 2015).

## Intestinal inflammation and barrier dysfunction

The intestinal mucosa is the inner lining of the GI tract. It is a physical and an immunological barrier between the environment and the internal host environs blood circulation, which is semipermeable and essential in the uptake of nutrients. It consists of an outer mucosal layer, a middle epithelial layer, and an inner lamina propria. The mucosal layer overlaying the epithelium is in close proximity to the external environment, communicates with gut microbiota, and keeps a check on pathogenic bacteria with immune sensing antimicrobial peptides and secretory IgA. A monolayer of epithelial cells with tight junction and adherens junction transport molecules and maintain the barrier’s integrity, respectively. Immune cells such as T-cells, B-cells, macrophages, and dendritic cells are in the lamina propria forming a defence barrier (Vancamelbeke and Vermeire 2017).

Intestinal bacteria and their metabolic by-products are one of the various factors contributing to the impairment of intestinal barrier function and hyperpermeability (Massier et al. 2021). Bacteria can damage the tight junction, alter the permeability and translocate through Peyer’s patches. Certain autoimmune disorders of the intestine can also trigger barrier dysfunction (Forsyth et al. 2011). Intestinal bacterial dysbiosis increases permeability and induces an intestinal inflammatory response, which activates neuroinflammation (Sun and Shen 2018). Researchers propose that TLRs are activated during dysbiosis and barrier dysfunction, which recognise bacterial lipopolysaccharides (LPS) presented by microglia and astrocytes. Thus, LPS hampers the intestinal barrier function and activates different immune cells, which in turn produce proinflammatory cytokines that cross the BBB and act on neurons and glial cells, leading to neuroinflammation and neuronal cell death (Sun and Shen 2018).

### Markers of intestinal inflammation and barrier dysfunction in PD

Investigation on the intestinal biopsies of the ascending colon has revealed intestinal inflammation is associated with higher concentrations of proinflammatory markers among PD cases (Devos et al. 2013). A study by Devos et al. on the stool immunological profile provides evidence of intestinal inflammation in PD, and their work also supports the association of intestinal dysfunction and altered microbiota in PD (Devos et al. 2013). The immune system can activate intestinal barrier dysfunction on exposure to bacterial endotoxins or environmental triggers leading to α-syn deposition, a hallmark of intestinal hyper-permeability in PD (Forsyth et al. 2011). Some of the studies have reported that the detection of calprotectin, a faecal marker of inflammation by ELISA, may be helpful in detecting early signs of the activated colonic immune system in PD (Schwiertz et al. 2018; Mulak et al. 2019). Calprotectin is secreted into the lumen by neutrophils on their migration to the inflammation site. Thus, calprotectin can serve as a faecal marker of inflammation as it can be found in subclinical inflammation and can resist degradation.

A protease inhibitor, alpha-1-antitrypsin concentrations are elevated in faeces of PD, indicating the damage in the GI barrier and the loss of proteins to the lumen. Similarly, zonulin modulates the tight junction and maintains intestinal barrier integrity. Alpha-1-antitrypsin and zonulin are markers of intestinal hyperpermeability in PD (Schwiertz et al. 2018). Though these markers are not specific to PD, they can be used as a non-invasive means for spotting intestinal inflammation and hyperpermeability in the early stage of the disease.

## Genetic predisposition and microbiome

Approximately 10 to 15% of PD are hereditary, and the rest is idiopathic. Multiple mutations and genes have been associated with PD. Most studies on gene mutation in PD are focused on LRRK2 (leucine-rich repeat kinase-2), PINK-1 (phosphatase and tensin [PTEN] homologue-induced kinase-1), and SNCA genes. Epigenetic alteration induced by exposure to the toxic chemical, environmental factors, pathogenic bacteria, or bacterial metabolites that interact with genes has been proposed to be involved in most sporadic PD cases (Kasper et al. 2014). LLRK2 gene has been linked to both PD and Crohn's disease (CD), an inflammatory bowel disease (IBD). Inflamed colonic tissue from PD and CD has higher levels of LRRK2, indicating the importance of the immune system in intestinal inflammation (Herrick and Tansey 2021). A systematic review and meta-analysis by Zhu et al. report a 28% and 30% increased risk of PD in patients with CD and ulcerative colitis, respectively (Zhu et al. 2019).

Genetic predisposition with microbiome interaction and association are sparsely studied in PD. SNCA is the α-syn encoding gene. Mutation in the SNCA gene affects its expression and is a risk factor for PD. SNCA has been associated with elevated opportunistic pathogens of the gut in PD. Certain bacterial species of the genus *Corynebacterium, Porphyromonas,* and *Prevotella* have been noted to be significantly abundant in PD and associated with SCNA genes (Wallen et al. 2021). These trios have been speculated as an opportunistic group with specific species of *C. amycolatum, C. lactis* of genus *Corynebacterium, P. asaccharolytica, P. bennonis, P. somerae, P. unonis* of genus P*orphyromonas and P. birria, P. buccalis, P. disiens, P. timonensis* of genus *Prevotella,* which are drastically altered in PD (Wallen et al. 2020, 2021). An overabundance of the unusually low *Corynebacterium* and endotoxins of the *Porphyromonas* and *Prevotella* can trigger the PD pathology in the gut (Wallen et al. 2021). Yet, there remains an enigma to solve and understand the underlying relationship between the gut microbiome and genetic predisposition in PD.

PINK1 and PRKN (parkin RBR E3 ubiquitin protein ligase) genes play a role in clearing damaged mitochondria. PINK1 and PRKN gene mutations are associated with mitochondrial dysfunction in the early-onset form of PD ((Kasper et al. 2014). Matheoud et al. have shown intestinal infection with Gram-negative bacteria in PINK1 − / − mice engage mitochondrial antigen presentation and autoimmune mechanisms to stimulate an inflammatory response in the periphery and the brain, triggering the PD-like motor symptoms in mice emphasising the gut-brain axis in the disease (Matheoud et al. 2019).

Peptidoglycan is a prominent cell wall component of the gram-negative bacterial cell. Microbes with peptidoglycan recognition protein encoding genes (PGLYRP) maintain a healthy gut by regulating immune responses to commensal and harmful bacteria. Variations in three PGLYRP genes of four have been linked with PD risk (Goldman et al. 2014).

## Gut microbiota associated with PD

**Fig. 2:**
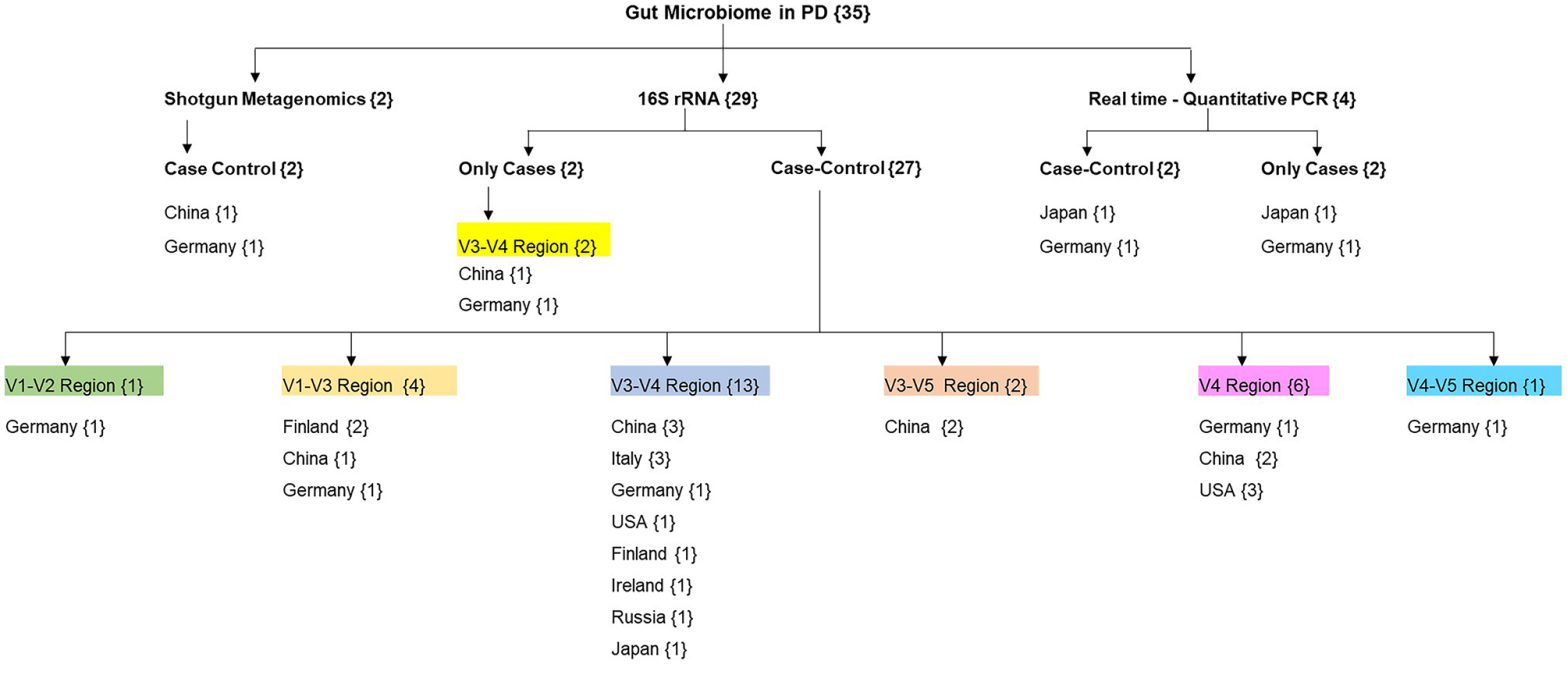
Studies on Gut Microbiome in Parkinson’s Disease. **Note:** Numbers mentioned in the {} are the number of articles. 16S rRNA gene codes for 30S subunit of bacterial ribosome. 16S rRNA gene sequencing aid in determining the bacterial diversity in a specific niche

Studies on the gut microbiome of PD have been carried out on diverse populations worldwide by following different protocols, most of which are case–control studies, a few are follow-up studies, some target a specific group of organisms, some others deal with shotgun metagenomics, and most of the others have employed 16S rRNA sequencing (Fig. 2). Various studies have analysed the relative abundance of bacterial taxa in PD and compared it with healthy controls (HC) using diverse platforms and databases. This review focuses on the intraluminal microbiome encountered in faecal samples of PD compared to HC, as stated in Table 1. Putative cellulose-degrading bacterial taxa *Prevotellaceae* (*Prevotella*)*, Ruminococcaceae* (*Faecalibacterium*)*, Lachnospiraceae* (*Blautia, Roseburia*) that produce SCFA and help in the synthesis of mucin to maintain the intestinal integrity are considerably lower in abundance in PD (Hill-Burns et al. 2017; Li et al. 2017; Aho et al. 2019; Cosma-Grigorov et al. 2020; Lubomski et al. 2021). On the contrary, a few putative pathobionts of the family *Enterobacteriaceae*, *Enterococcaceae*, which are assumed to possibly reduce the production of SCFA, produce endotoxins and neurotoxins that promote intestinal inflammation, are enriched in PD (Li et al. 2017; Barichella et al. 2019). An altered microbial composition, a decrease in bacteria associated with SCFA synthesis that is bacteria related to anti-inflammation, and a higher abundance of proinflammatory pathobionts of phylum Proteobacteria in PD, are similar to that of the changes observed in IBD (Keshavarzian et al. 2015).

**Table 1:** Differential Abundance of Microbiota Observed in PD vs. HC

	**Phylum**	**Family**	**Genus**
**More Abundant in PD**	Verrucomicrobia {7}	*Verrucomicrobiaceae* {9}	*Akkermansia* {14}
	Actinobacteria {2}	*Bifidobacteriaceae* {6}	*Bifidobacterium* {6}
	Proteobacteria {2}	*Enterobacteriaceae* {4}	*Klebsiella* {3}
		*Desulfovibrionaceae* {2}	*Bilophila* {3}
		*Moraxellaceae* {1}	*Acinetobacter* {2}
	Bacteroidetes {2}	*Porphyromonadaceae* {3}	*Porphyromonas* {1}
		*Rikenellaceae* {3}	*Alistipes* {3}
		*Bacteroidaceae*	*Bacteroides* {2}
		*Tannerellaceae* {3}	*Parabacteroides* {3}
	Firmicutes {4}	*Lactobacillaceae* {9}	*Lactobacillus* {4}
		*Christensenellaceae* {6}	*Christenella* {4}
		*Clostridiaceae* {4}	*Anaerotruncus* {3}
		*Clostridiaceae* {4}	*Clostridium* {2}
		*Ruminococcaceae* {4}	*Oscillospira* {2}
		*Enterococcaceae* {3}	*Enterococcus* {2}
		*Streptococcaceae*	*Streptococcus* ↑ {2}
		*Veillonellaceae* {3}	*Veillonella* {1}
**Less Abundant in PD**	Bacteroidetes {3}	*Prevotellaceae* {9}	*Prevotella* {5}
	Firmicutes {2}	*Ruminococcaceae* {2}	*Faecalibacterium* {8}
		*Lachnospiraceae* {7}	*Blautia* {7}
			*Roseburia* {7}
			*Fusicatenibacter* {3}
			UC *Lachnospiraceae* {3}
			*Dorea* {2}
			*Coprococcus* {2}
	Proteobacteria	*Pasteurellaceae* {4}	*Haemophilus* {2}

*HC* Healthy control, *UC* Unclassified, Numbers mentioned in the {} are the number of articles reporting the abundance

Mucin degrading genus *Akkermansia* of the phylum Verrucomicrobia has been widely reported to be significantly abundant in PD by most studies. *Akkermansia* and *Christensenellaceae* may symbiotically play a role in PD pathology and progression (Nishiwaki et al. 2020). Intestinal mucus layer is rich in protein mucin. *Akkermansia* utilises mucin as a nutritional source and degrades it into SCFA acetate, which acts as a substrate for other beneficial bacteria to produce butyrate, an energy source for the intestinal epithelial cells (Belzer et al. 2017). *Akkermansia* is a symbiont that degrades mucin and encourages cells to produce more mucin (Derrien et al. 2017) and, in turn, enhances the mucosal integrity and modulates the immune system. If the intestinal cells fail to produce mucin, it would eventually lead to detrimental effects, a leaky gut and inflammation. A few studies that reported a lower abundance of putative complex cellulose-degrading bacteria and have also found a higher abundance in *Akkermanisa* (Hill-Burns et al. 2017; Cirstea et al. 2020; Vidal-martinez et al. 2020). A compensatory effect of richness in *Akkermansia* is possibly due to depleting cellulose-degrading bacteria in the PD gut.

Various studies have also reported a correlation in the abundance of bacterial taxa with respect to disease duration, disease severity, motor symptom score, non-motor symptoms, particularly constipation, and antiparkinsonian treatment in PD (Table 2). Bradykinesia, rigidity, resting tremor, and gait impairment are cardinal motor symptoms of Parkinson’s that aid in the diagnosis and prognosis of the disease. Unified Parkinson’s disease rating scale (UPDRS) is broadly used to evaluate the disease state, and UPDRS part III is specific for motor symptoms (Goetz et al. 2007). An abundance of specific microbiota has been linked with motor impairment, as mentioned in Table 2. *Aquabacterium*, *Peptococcus*, and *Sphingomonas* are associated with motor complications in PD (Qian et al. 2018). Keshavarzian et al. have reported a higher abundance of phyla Proteobacteria and a lower abundance of Firmicutes with PD duration (Keshavarzian et al. 2015). Keshavarzian et al. and Hill-Burns et al. found *Lachnospiraceae* to be negatively correlated with duration (Keshavarzian et al. 2015; Hill-Burns et al. 2017), although Barichella et al. reported reduced levels of *Lachnospiraceae* in all duration of PD (Barichella et al. 2019). Barichella et al. have also observed an effect of disease duration on microbiota, indicating increasing levels of family *Lactobacillaceae* and a co-abundant genus *Akkermansia* (Barichella et al. 2019). Hasegawa et al. suggest an increase in *Lactobacillus gasseri* subgroup can predict disease duration in PD (Hasegawa et al. 2015). Lin et al. observed *Pasteurellaceae, Alcaligenaceae, and Fusobacteria* were more abundant in the early onset of PD, whereas *Comamonas* and *Anaerotruncus* were abundant in the late onset of PD (Lin et al. 2018).

**Table 2:** Microbiota Associated with PD

Microbiota			Motor—UPDRS III	Non-Motor Symptoms	Severity	Duration	Constipation	iCOMT	Levodopa
**Family**	**High**	*Enterobacteriaceae*	(Pietrucci et al. 2019)	-	(Barichella et al. 2019; Pietrucci et al. 2019)	(Li et al. 2017; Melis et al. 2021))	-	(Scheperjans et al. 2015; Lin et al. 2018; Ren et al. 2020; Melis et al. 2021)	-
		*Lactobacillaceae*	(Barichella et al. 2019)	(Barichella et al. 2019; Jin et al. 2019)	-	(Hasegawa et al. 2015; Barichella et al. 2019)	(Nishiwaki et al. 2020)	(Barichella et al. 2019)	-
		*Ruminococcaceae*	(Hill-Burns et al. 2017; Lin et al, 2018)	-	-	(Hill-Burns et al. 2017; Lin et al. 2018; Hegelmaier et al. 2020)	-	(Barichella et al. 2019)	-
	**Low**	*Lachnospiraceae*	(Barichella et al. 2019; Pietrucci et al. 2019)	(Barichella et al. 2019)	-	(Keshavarzian et al. 2015; Hill-burns et al. 2017)	(Nishiwaki et al. 2020)	(Hill-burns et al. 2017; Barichella et al. 2019)	-
		*Prevotellaceae*	(Scheperjans et al. 2015)	-	-	-	-	-	-
**Genus**	**High**	*Akkermansia*	-	(Heintz-Buschart et al. 2018)	-	(Hill-burns et al. 2017; Barichella et al. 2019)	(Heintz-Buschart et al. 2018; Baldini et al. 2020; Lubomski et al. 2021; Melis et al. 2021)	-	-
		*Bifidobacterium*	-	(Qian et al. 2018)	-	-	(Baldini et al. 2020)	(Lin et al. 2018; Aho et al. 2019; Weis et al. 2019))	-
		*Eubacterium*	-	-	(Jin et al. 2019)	-	-	(Weis et al. 2019)	-
		*Enterococcus*	(Li et al. 2017)	(Jin et al. 2019)	(Li et al. 2017)	(Li et al. 2017)	-	-	(Weis et al. 2019)
	**Low**	*Blautia*	(Li et al. 2017)	(Melis et al. 2021)	(Li et al. 2017)	-	-	(Hill-Burns et al. 2017)	(Melis et al. 2021)
		*Faecalibacterium*	(Li et al. 2017)	-	(Pietrucci et al. 2019; Weis et al. 2019)	(Weis et al. 2019; Cosma-Grigorov et al. 2020)	(Cirstea et al. 2020; Nishiwaki et al 2020)	(Weis et al. 2019)	(Weis et al. 2019; Melis et al. 2021)
		*Roseburia*	-	-	-	-	(Cirstea et al. 2020)	-	(Melis et al. 2021)

Higher abundance of *Proteus*, and *Escherichia*-*Shigella* of family *Enterobacteriaceae* have been observed with enhanced motor symptoms. Bacteria associated with SCFA metabolisms such as family *Lachnospiraceae*, *Prevotellaceae* genus *Blautia*, and *Faecalibacterium* are lower in abundance with worsening motor symptoms. An abundance of Genus *Akkermansia* (*Verrucomicrobiaceae*, Verrucomicrobia), *Escherichia-Shigella*, *Proteus*, *Enterococcus* (pathobionts), *Lactobacillus* (*Lactobacillaceae*) and family *Ruminococcaceae* are positively correlated and Cellulose degrading bacteria *Blautia* and *Faecalibacterium* are negatively correlated with disease duration

*UPDRS III* Unified Parkinson’s Disease Rating Scale III for Motor Examination, *iCOMT* Catechol-O-methyltransferase inhibitors

Some studies have also noted variation in microbial diversity between tremor dominant and non-tremor PD subjects. *Roseburia* (Lin et al. 2018), *Flavobacterium*, *Bacteroidia*, *Propionibacterium*, and *Alcaligenaceae* (Lin et al. 2019) are abundant in non-tremor subjects. *Leptotrichia* (Lin et al. 2018), *Clostridium*, *Verrucomicrobia*, and *Akkermansia* (Lin et al. 2019) are abundant in tremor dominant PD subjects, while the family *Ruminococcaceae* (Weis et al. 2019) is low. Increased levels of *Lactobacillaceae* (Barichella et al. 2019) and *Enterobacteriaceae* (Scheperjans et al. 2015; Lin et al. 2018; Pietrucci et al. 2019) but a reduced level of *Lachnospiraceae* (Barichella et al. 2019) is associated with postural instability and gait disturbance in PD. Reduced abundance of genus *Prevotella* in patients with faster disease progression (Aho et al. 2019) has been observed. Symbiotically *Akkermansia* and *Christensenellaceae* are predicted to play a role in the advancement of PD (Nishiwaki et al. 2020). Hill-Burns et al. describe family *Ruminococcaceae* increases in abundance as a consequence of disease duration as they observed *Ruminococcaceae* not to be high in patients within the first ten years of the disease but to be highly elevated in patients with the disease for more than ten years (Hill-Burns et al. 2017).

Anosmia, sensory disturbance, mood disorders, sleep disturbance, and autonomic disturbance are additional pre-motor and non-motor features in PD. Barichella et al. observed increased levels of *Lactobacillaceae* and reduced *Lachnospiraceae* and related genera with intellectual impairment (Barichella et al. 2019). A study by Rem et al. have analysed the gut microbiota in PD with cognitive impairment by employing the mini-mental state examination (MMSE) and Montreal cognitive assessment (MoCA) questionnaires. Genus *Alistipes* and *Odoribatcer* are negatively correlated with MoCA scores. *Barnesiella* is negatively associated with MMSE scores. *Butyricimonas* have been negatively correlated with MMSE and MoCA scores. Genus *Blautia* of the family *Lachnospiraceae* was observed to be depleted in patients with mild cognitive impairment, whereas family *Rikenellaceae* and *Ruminococcaceae* were rich (Ren et al. 2020). Reduced abundance of *Bifidobacterium* is associated with depression in PD (Qian et al. 2018).

GI dysfunction is one of the autonomic disturbances in PD. A range of PD-associated GI dysfunctions has been clinically identified, involving weight loss, gastroparesis, constipation, and defecation dysfunction (Kim and Sung 2015). James Parkinson has mentioned constipation as one of the symptoms experienced by his first shaking palsy subject, which is now attributed as one of the prodromal non-motor symptoms in PD (Parkinson 2002). Constipation has been reported in approximately 60 percent of patients with PD (Kaye et al. 2006). Recent research has indicated that gut microbiota may contribute to constipation and related symptoms (Zhao and Yu 2016). A richness of *Lactobacillaceae* (Nishiwaki et al. 2020), *Verrucomicrobiaceae, Bradyrhizobiaceae* (Scheperjans et al. 2015), *Bifidobacterium* (Baldini et al. 2020), and *Akkermansia* (Heintz-Buschart et al. 2018; Baldini et al. 2020; Cirstea et al. 2020; Lubomski et al. 2021), and lower abundance of *Lachinospiraceae* (Nishiwaki et al. 2020), *Roseburia* (Cirstea et al. 2020), and *Faecalibacterium* (Cirstea et al. 2020; Nishiwaki et al. 2020) have been correlated with constipation or bristol score in PD. *Akkermansia* has been associated with slow transit time (Heintz-Buschart et al. 2018; Baldini et al. 2020), firmness of stool (Cirstea et al. 2020), and constipation severity (Lubomski et al. 2021).

## Antiparkinsonian treatment

Therapeutic drugs targeting humans affect the gut microbiome, which is also relevant the other way round, referred to as bacteria-drug interactions. Gut bacteria render the drug less available for the target, either by bioaccumulation and biotransformation or biodegradation. Intestinal bacteria store drugs intracellularly during bioaccumulation without modifying the chemical, where bacterial growth remains unaffected. In biotransformation, bacteria chemically metabolise the drug by their enzymatic secretions, making the drug ineffective and altering the microbial community composition by metabolic cross-feeding (Klünemann et al. 2021).

Various studies describe the different impacts of antiparkinsonian drugs on the gut microbiome. Different classes of drugs are employed in PD treatment. Some studies examined the impact of dopamine therapy on microbial composition and reported a lower abundance of *Faecalibacterium*, an anti-inflammatory SCFA producing bacteria (Weis et al. 2019; Melis et al. 2021). Several studies have reported a strong impact of the catechol-O-methyltransferase inhibitors (iCOMT) on microbial diversity and compositions and are considered as confounding factors while analysing the PD gut microbiome and confirmed by metanalysis (Nishiwaki et al. 2020). The richness of *Enterobacteriaceae* has been significantly associated with iCOMT (Scheperjans et al. 2015; Lin et al. 2018; Ren et al. 2020; Melis et al. 2021), and *Bifidobacterium* is more common in iCOMT users (Lin et al. 2018; Aho et al. 2019; Weis et al. 2019). However, *Lachnospiraceae* has been noted to be lower in abundance (Hill-Burns et al. 2017; Barichella et al. 2019).

Two studies have analysed the influence of device-assisted therapy in PD on microbial composition, and both studies describe the over-representation of *Enterobacteriaceae* in PD subjects on the levodopa-carbidopa intestinal gel (LCIG) (Lubomski et al. 2021; Melis et al. 2021). The abundance of *Enterobacteriaceae* has been assumed as a result of inflammation by LCIG usage (Melis et al. 2021). An abundance of *Clostridium cluster XlVa*, *Bilophila*, *Parabacteroides*, and *Pseudoflavonifractor* and a lower abundance of *Dorea* have been reported in patients on deep brain stimulation (DBS) therapy (Lubomski et al. 2021).

### Microbes associated with PD drug metabolism

*Helicobacter pylori*, the causative organism of peptic ulcers, has been associated with different GI infections. *H pylori* infection is correlated with motor fluctuations, impaired levodopa absorption with worsening symptoms, and aggravating neurodegenerative process in PD (Pierantozzi et al. 2006; Tan et al. 2014). Randomised control trials on eliminating *H. pylori* infection have generated conflicting results. Lolekha et al. report a decrease in wearing off symptoms post medication and significant clinical improvement in the symptoms (Lolekha et al. 2021), contradicting the study by Tan et al. that did not notice any substantial changes in the symptoms (Tan et al. 2020). Recently *Clostridium sporogenes* have been associated with deamination of unabsorbed residual levodopa in the intestine to 3-(3,4-dihydroxyphenyl) propionic acid, which has been shown to exert an inhibitory effect on ileal motility in an ex-vivo model and speculated to increase the transit time (Van Kessel et al. 2020).

Levodopa, a primary drug for the treatment of PD, is absorbed in the jejunum (Gundert-Remy et al. 1983). Levodopa must enter the brain and be converted to the neurotransmitter dopamine by the human enzyme aromatic amino acid decarboxylase (AADC) in order to be effective. Intestinal bacteria, *Enterococcus faecalis,* can convert levodopa to dopamine in the gut before crossing the BBB rendering the drug ineffective as dopamine is incapable of crossing the BBB. Rekdal et al. demonstrated that *Enterococcus faecalis* and *Eggerthella lenta* metabolise levodopa sequentially to m-tyramine employing the enzymes tyrosine decarboxylase and dopamine decarboxylase, respectively, affecting the pharmacokinetics of the drug. They have also discovered that intestinal bacterial levodopa decarboxylation can be inactivated by (*S*)-α-fluoromethyltyrosine (AFMT) (Rekdal et al. 2019).

A rat in situ study model discovered the presence of bacterial tyrosine decarboxylase in the small bowel contributes to interindividual variation in drug efficacy and a higher abundance of the microbial tyrosine decarboxylase gene in the small bowel of rats diminishes the levels of levodopa in small intestinal and plasma. The same study has also found a positive correlation between higher bacterial tyrosine decarboxylase gene in PD faecal samples with disease duration and levodopa daily dose (Van Kessel et al. 2019). This may possibly explain how some PD patients require a higher dosage regimen of levodopa treatment.

## Intestinal bacterial metabolites

Bacteria inhabiting the gut metabolise carbohydrates and proteins to SCFA and amino acids to acquire energy and proliferate. SCFA, such as butyrate, propionate, and acetate, in addition to amino acids such as p-cresol and phenylacetylglutamine, are generated on carbohydrate and protein metabolism by intestinal bacteria (Oliphant and Allen-Vercoe 2019). Recent research has established that some GI abnormalities are contributed by or related to changes in the composition of the gut microbiota and their metabolites (Zhao and Yu 2016). The gut microbiome analysis revealed that the PD microbiota composition, function, and metabolic pathways are skewed toward proteolytic metabolism, which is linked with GI dysfunction in PD (Cirstea et al. 2020).

Microbiota responsible for nucleic acid and amino acid metabolism pathways is enhanced in PD subjects, whereas microbiota carrying out the carbohydrate degradation pathway are abundant in healthy subjects (Cirstea et al. 2020). Unger et al. confirmed a significant reduction in the SCFA in the faeces of PD subjects, consistent with a lower abundance of SCFA producing bacteria and suggesting it can contribute to constipation in PD. SCFA are produced as a metabolic product of complex carbohydrate metabolism by the beneficial gut microbiota (Unger et al. 2016). SCFAs are signalling molecules with anti-inflammatory and antioxidant properties (Huuskonen et al. 2004). Depleting SCFA in the intestine results in devastating effects on the GI barrier function with enhanced inflammation and intensifies the risk of α-syn deposition and brain microglial activation (Mulak 2018).

Melis et al. showed altered microbial composition matched with metabolic changes affecting the metabolism of lipids and proteins. They report an upsurge in metabolites cadaverine and putrescine with proinflammatory activity in PD subjects. They have found an abundance of 4-hydroxyphenylpropionic acid in patients on levodopa therapy and speculate the existence of numerous metabolic pathways to degrade the drug (Melis et al. 2021). Cristea et al. have noted enriched fucose degradation, which possibly indicates host mucin breakdown. They observed elevated levels of para-cresol and phenylacetylglutamine and their association with stool consistency (Cirstea et al. 2020).

## Diet and microbiome in PD

Diet is the most important factor determining the composition and function of the microbiome and its metabolites in the gut (Heiman and Greenway 2016; Jackson et al. 2019). Change in diet can alter the gut microbial profile of an individual, which can be transient if the modifications are for a short duration or if the amendment is continued for an extended period, the established bacteria will be replaced according to the diet. However, it would revert to the original composition once the dietary modifications are terminated (Wu et al. 2011; David et al. 2014). Consumption of an animal-based diet increases the abundance of bile-tolerant microorganisms and lowers the mass of Firmicutes that utilize dietary plant polysaccharides (David et al. 2014). Bacteroides enterotypes are associated with protein and animal fat consumption (Wu et al. 2011). A plant-based diet that is a higher intake of fibres is associated with increased production of SCFA and positively correlated with levels of *Prevotella* in the gut (Wu et al. 2011; David et al. 2014; De Filippis et al. 2016).

Derkinderen et al. postulate that reduced risk of PD with cigarette smoking and coffee consumption may be associated with shaping the gut microbial composition that exhibits anti-inflammatory properties by their metabolites that reduces intestinal inflammation (Derkinderen et al. 2014). In a dietary intervention by adding whole-grain barley and brown rice to the diet, Martinez et al. report increased microbial diversity and immunological and metabolic improvement (Martínez et al. 2013). Two studies on the American and Greece populations have revealed that individuals on a strict Mediterranean diet have a reduced risk of prodromal PD symptoms (Maraki et al. 2019; Molsberry et al. 2020). A survey by Metcalfe-Roach et al. suggests the Mediterranean diet can be a nutritional strategy to delay the onset of PD (Metcalfe-Roach et al. 2021).

## Prebiotic, probiotic, and faecal microbiome transplantation approach in PD therapeutics

As reported in the review, studies have demonstrated altered gut microbiome is potentially associated with PD pathology and outcome. Faecal microbiome transplantation (FMT), prebiotics, and probiotics are potential options for restoring the altered microbiome in PD. An interventional study on a small group of PD subjects by Hegelmaier et al. suggests the inclusion of dietary SCFA has a positive influence on the gut microbiome and has a positive outcome on the course of the PD (Hegelmaier et al. 2020). Similarly, prebiotic intervention with the inclusion of resistant starch in the diet resulted in a decline in non-motors symptoms, stabilised faecal microbial diversity, improved butyrate levels, and reduced calprotectin concentration in PD (Becker et al. 2021). In clinical trials, increase in spontaneous bowel movements in PD patients with constipation (Ibrahim et al. 2020; Tan et al. 2021), an improvement in abdominal pain and bloating (Georgescu et al. 2016), and a decrease in UPDRS score (Tamtaji et al. 2019) has been observed on using different strains of multi-strain probiotics. A randomized controlled trial showed the intake of fermented milk with prebiotic fibers and different probiotic strains (*Lactobacillus spp*, *Bifidobacterium spp*, *Streptococcus salivarius*, and *Enterococcus faecium*) significantly increased the frequency of complete bowel movements in PD patients improving constipation (Barichella et al. 2016). Combination therapy is the only treatment recommended as “efficacious” and “clinically useful” by the latest MDS evidence-based guidelines (Seppi et al. 2019).

Following the significant success in treating recurrent *Clostridium difficile* infections with FMT (Silverman et al. 2010), scientists have focused on treating non-curable diseases by modifying the gut microbiome (Merenstein et al. 2014). FMT involves the transfer of screened stool that contains microbes and their metabolites from a healthy donor to a patient (Merenstein et al. 2014). Some of the studies have thrived on revealing the practical implication of FMT in PD. Both Xue et al. and Kuai et al. report FMT improves motor and nonmotor symptoms in PD (Xue et al. 2020; Kuai et al. 2021). Xue et al. compared FMT via the colonoscopy and nasointestinal tube and recommend colonoscopy over the latter since it had a relatively more satisfactory impact on PD symptoms, improving anxiety, depression, and sleep quality (Xue et al. 2020). Kuai et al. have analysed the gut microbiome of PD subjects before and after FMT and report an increased abundance of *Blautia*, *Lachnospiraceae*, and *Prevotella* in PD, whereas a decrease in abundance of Bacteroidetes post-FMT (Kuai et al. 2021). Recovery in GI symptoms and remission in constipation was documented after FMT therapy in PD subjects with constipation (Huang et al. 2019; Kuai et al. 2021). Further confirmatory studies are essential on the application of prebiotics, probiotics, and FMT as therapeutics in PD, which may also be personalised as per the requirements of the patients.

A recent intriguing study has brought together a new hypothesis of brain-first PD centered on prodromal rapid eye movement sleep behaviour disorder conflicting with the existing body-first PD. This Danish research reports two PD subtypes based on the initial starting point and spread of α-syn pathology: brain-first PD, neuropathology begins in the brain and then spreads downward, while other being the body-first PD subtype, as suggested by Braak that PD pathology originates in the body from GI and olfactory peripheral nervous systems and ascends towards the brainstem (Horsager et al. 2020). Their investigation has opened a new horizon for microbiome scientists to explore and understand the microbial diversity in the two subtypes. Microbes and their metabolites may vary in PD subtypes and even have a differential impact on the disease progression. Since none of the studies have considered the possibilities of the different microbial compositions within PD or have grouped them based on the origin of the synucleinopathy, any interventions aimed to modify the gut microbiota would be ineffective if the disease started in the brain. It would be relevant for the researchers to look into the microbiome involvement in the different subtypes and stages of PD for effective personalised therapy.

## Conclusion

In recent years, there has been an increased interest in discovering the genesis of PD and identifying markers that can assist in detecting the condition early to tailor treatment strategies accordingly. Despite this, none of the research covered in this review has consistently predicted the common microbial signature associated with PD, and it could be the result of the brain-first and body-first PD subtypes. In general, the negative impact of the imbalance in the bacterial community that promotes inflammatory response and protective bacterial communities producing SCFA has been documented in PD. Gut microbiota and their metabolic products are associated with motor and non-motor symptoms of PD, with genetic predisposition, and with antiparkinsonian drug metabolism. However, the findings are quite heterogeneous across the studies due to differences in the study population and methodology. Therefore, we should move beyond microbiome composition and potential metabolic entities to direct the evaluation of active metabolic pathways. A multi-omics approach is likely required to understand the function of the gut microbiota in PD progression for better diagnosis and treatment. Dietary and microbial interventions are promising stratagems for disease modulation and therapy. Additional clinical trials on personalised FMT and nutritional modifications in PD would probably enhance our knowledge of the gut microbiota and brain interactions for better treatment approaches.
